# Risk Factors for and Clinical Outcome of Congenital Cytomegalovirus Infection in a Peri-Urban West-African Birth Cohort

**DOI:** 10.1371/journal.pone.0000492

**Published:** 2007-06-06

**Authors:** Marianne A.B. van der Sande, Steve Kaye, David J.C. Miles, Pauline Waight, David J. Jeffries, Olubukola O. Ojuola, Melba Palmero, Margaret Pinder, Jamila Ismaili, Katie L. Flanagan, Akum A. Aveika, Akram Zaman, Sarah Rowland-Jones, Samuel J. McConkey, Hilton C. Whittle, Arnaud Marchant

**Affiliations:** Medical Research Council Laboratories, Fajara, The Gambia; US Naval Medical Research Center Detachment/Centers for Disease Control, United States of America

## Abstract

**Background:**

Congenital cytomegalovirus (CMV) infection is the most prevalent congenital infection worldwide. Epidemiology and clinical outcomes are known to vary with socio-economic background, but few data are available from developing countries, where the overall burden of infectious diseases is frequently high.

**Methodology/Principal Findings:**

As part of an ongoing birth cohort study in The Gambia among term infants, urine samples were collected at birth and tested by PCR for the presence of CMV DNA. Risk factors for transmission and clinical outcome were assessed, including placental malaria infection. Babies were followed up at home monthly for morbidity and anthropometry, and at one year of age a clinical evaluation was performed. The prevalence of congenital CMV infection was 5.4% (40/741). A higher prevalence of hepatomegaly was the only significant clinical difference at birth. Congenitally infected children were more often first born babies (adjusted odds ratio (OR) 5.3, 95% confidence interval (CI) 2.0-13.7), more frequently born in crowded compounds (adjusted OR 2.9, 95%CI 1.0-8.3) and active placental malaria was more prevalent (adjusted OR 2.9, 95%CI 1.0-8.4). These associations were corrected for maternal age, bed net use and season of birth. During the first year of follow up, mothers of congenitally infected children reported more health complaints for their child.

**Conclusions/Significance:**

In this study, the prevalence of congenital CMV among healthy neonates was much higher than previously reported in industrialised countries, and was associated with active placental malaria infection. There were no obvious clinical implications during the first year of life. The effect of early life CMV on the developing infant in the Gambia could be mitigated by environmental factors, such as the high burden of other infections.

## Introduction

Congenital cytomegalovirus (CMV) infection is the most prevalent congenital infection worldwide, with recent estimates ranging from 0.1–2% of all pregnancies being affected [1]–[4]. Infection-in-utero is potentially fatal to the foetus, and is associated with a range of adverse outcomes involving multiple organs, in particular the liver and the central nervous system. Of those born with congenital infection, up to 10% of infected foetuses have mild or severe disease. Neonatal mortality associated with symptomatic congenital CMV infection during the first year of life is estimated to be over 10% [5]. The majority of those with a congenital infection are however asymptomatic at birth. Of those born without obvious clinical symptoms, another 10% may suffer from long-term sequelae, in particular audito-neurological complications, with hearing loss being the most prevalent [6], [7].

Congenital CMV infection occurs through vertical transmission of the virus by an infected pregnant woman to the foetus via the placenta. This may arise through primary infection of the mother, reactivation during pregnancy of a latent infection or re-infection with a different strain of CMV [8]. The incubation period of the infection is about 2–3 weeks, after which shedding of the virus can be detected in secretions such as urine, vaginal secretions and breast milk [9]. Transmission occurs through shedding of the virus in body fluids during such periods of active replication, although the mechanisms involved in transplacental transfer remain poorly understood [10], [11]. In the industrialised world, where prevalence rates among young adults are estimated to be around 40–50%, congenital infection usually occurs following a primary infection of the mother during pregnancy [12]. Such a primary infection is estimated to occur in 1–4% of pregnancies, 20 to 40% of which subsequently result in transmission of CMV to the foetus. Clinical symptoms tend to be less severe among children infected following reactivation of the virus during pregnancy. Nevertheless, recent data from industrialised countries suggest that congenital infections following recurrent maternal infections may represent a significant proportion of the disease burden associated with congenital CMV infection [13], [14].

CMV infection has a profound impact on the immune system by decreasing cell-mediated immune responses during the early phase of the infection and by promoting immune ageing [15], [16]. We have shown that CMV infection in early life induces a large subset of T lymphocytes expressing a late differentiation phenotype [17], that is associated with immunosenescence in the elderly [18], [19]. Therefore, congenital CMV infection may not only lead directly to increased morbidity at birth and during the early years, but could also impact indirectly on the health of an infected child through immunosuppression. This could interfere with a robust response on the routine infant vaccinations, and with the response to other infections.

Clinical and epidemiological patterns of CMV infection are known to differ according to socio-economic and geographical settings, although there are limited data from developing countries. We studied the incidence and clinical sequelae of congenital CMV within a peri-urban birth cohort, and explored the impact of a range of risk factors (placental malaria, CMV viral load, maternal and infant demographics) on placental transmission and clinical outcome. We hypothesised that placental malaria and CMV maternal viral load would contribute to the risk of congenital infection, and that apparently asymptomatic infection might still affect the clinical development and health of the infants.

We here report on risk factors for congenital CMV infection in a highly endemic region and the clinical impact of a congenital infection observed during the first year of life in a cohort of term, healthy, babies in The Gambia.

## Methods

### Subjects

In January 2002, a birth cohort was initiated in the village of Sukuta, The Gambia, approximately 15 km away from the Medical Research Council (MRC) Laboratories in Fajara, in order to study the epidemiological, clinical, immunological and virological determinants of early life CMV infection in an endemic environment. The wider objective of this cohort was to understand how the immune system of the foetus and new born develops in relation to CMV and other early life infections, and how this interacts with the response to vaccinations.

Sukuta has approximately 25,000 inhabitants, and is adjacent to, but distinct from the nearby expanding peri-urban community centred around the village of Serrekunda between the capital Banjul 20 km to the north and a growing urban centre (Brikama) 25 km to the south. A variety of ethnic groups live in the village, although the Mandinka group make up about half of the population.

Eligible for recruitment in the cohort were children born in the health centre of the study village whose parents gave informed consent. Babies whose mothers had suffered a serious infectious disease during pregnancy for which they had been admitted were not recruited. Pre-term babies, babies born with a serious congenital deficit, or babies who were in need for a transfer to a referral hospital immediately following delivery, were not included either. Routine follow up was conducted monthly. During these routine visits, anthropometric measurements and a morbidity checklist were completed. Vaccinations were given according to the Gambia Government schedule. Recruitment and follow-up are still ongoing.

### Laboratory investigations

#### a. CMV

Urine was collected within two weeks of birth and transported to the laboratory within 24 hours, and stored at −20°C. CMV DNA was detected by an in-house nested PCR method amplifying a region of the UL50 gene. Frequent negative controls (one in seven reactions) were included to minimise the risk of false-positive reactions. CMV viral load in maternal samples collected at the time of delivery (vagina, urine, saliva, colostrum and plasma) from a sub-set of mothers was quantified by real-time PCR. The lower limit of detection by real-time PCR was 100 copies/ml for plasma and vaginal swab, and 50 copies/ml for the other samples. Maternal plasma levels of CMV-specific IgG were detected and quantified using commercial enzyme immunoassays (DiaSorin, Saluggia, Italy) used according to the manufacturer's instructions. The complete results from maternal testing will be described in a subsequent viroimmunology paper.

#### b. Placental malaria

Immediately upon delivery, a placental imprint was made on a glass slide. Slides were Giemsa stained, and transported to the laboratory for the detection of parasitaemia. Slides were read by a trained microscopist for the speciation and quantification of malaria parasites; and a random selection of slides was read by a second trained microscopist to assure quality control. At least 100 fields were read before a negative result was declared. A placental biopsy was taken and immediately placed into 10% formalin for transport to the laboratory. Each placental sample was embedded in paraffin wax, sectioned and stained with standard H&E stain. Slides were examined under a light microscope for evidence of malaria infection according to the classification described by Ismail *et al*. [20]. Thus placentas were ascribed to one of 4 groups: no infection, acute infection (parasites in the intervillous space), chronic infection (parasites and malaria pigment) or past infection (pigment only).

### Clinical evaluation

A paediatric assessment, including a neurological examination of the child was performed at birth by a qualified paediatrician (first OO, later MP), at which time the CMV status of the baby was not yet known. Baseline data were collected at birth, and every month, morbidity data and anthropometry was collected in a standardised way. Parents could consult the paediatrician with any complaints or concerns about their children's health and welfare throughout the follow up period, and at one year of age another standardised clinical evaluation was performed by the paediatrician. Maternal height and weight were measured six months after delivery.

### Definitions

Congenital CMV was defined as the detection of CMV in the urine by PCR within two weeks of birth. Acute malaria infection was defined as the detection of malaria parasites by microscopy, or the detection of an acute infection by histology. Active malaria infection was defined as a histological diagnosis of acute or chronic infection.

### Analysis

Field, clinic and laboratory data were all merged and validated in a relational Microsoft Access database. Data were analysed using Stata 8 (Stata Corp, Texas, USA). Statistical significance was assigned when a p-value<0.05 was obtained. Differences in proportions were compared with a chi-squared test. Univariate logistic regression analysis was used to calculate odds ratio's (ORs) to test for significance of associations between risk factors and congenital CMV infection. Where needed, variables were dichotomised or categorised to enable inclusion in a logistic regression analysis. Risk factors were included in a stepwise backward multivariate model if p<0.1 to obtain independent adjusted ORs.

### Ethics

The study was approved by the Gambia Government/MRC Ethics Committee. All parents gave written informed consent for their child to participate.

## Results

### Subjects

Between January 2002 and January 2005, 840 eligible babies were recruited into the cohort. Urine was collected within the first two weeks of life from 741 of them (88.2%), and congenital CMV was diagnosed in 40/741 newborns (5.4%). The prevalence of congenital infection was higher among first born babies (11.2% vs 3.8%, p-value<0.001). If a baby was not a mother's first child, then congenital infection was associated with being born into a larger family (4.1 older siblings vs 3.1, p-value = 0.04). Although not statistically significant, babies with CMV were born into slightly more populated compounds (table 1).

**Table 1:** Background characteristics of babies born with and without congenital CMV infection recruited January 2002 to January 2005.

	Congenital CMV infection	No congenital CMV infection	p-value
N	40 (5.4)	701 (94.6)	
n (%) females	20 (50.0)	330 (47.1)	0.7
Maternal age (yrs, median, IQR)	23 (20–30)	25 (21–30)	0.3
Maternal body mass index (m^2^/(kg) (mean, sd)	21.5 (4.8)	22.2 (4.1)	0.5
n (%) first pregnancy	18 (46.2)	143 (20.5)	<0.001
If not first child:			
number of elder siblings alive (mean, sd)	3.6 (2.5)	2.7 (1.8)	0.02
n/N (%) where elder sib died	9/22 (40.9)	166/551 (32.0)	0.4
People living in same compound (median, IQR)	16 (10–26)	13 (8–20)	0.1
Placental parasitaemia (n/N, %)	6/24 (25.0)	50/445 (11.2)	0.04
Acute placental malaria infection (n/N, %)	7/23(30.4)	63/413 (15.3)	0.05
Born in malaria/hungry [41] season (August-December) (n, %)	16 (40.0)	326 (46.5)	0.4

IQR = inter quartile range; sd = standard deviation

### At Birth

Among the congenitally infected group, the prevalence of first pregnancies was significantly higher (50% vs 21%, p<0.001). There was no seasonal variation in the detection of congenital CMV infection, but the prevalence of placental parasitaemia was significantly higher among cases of congenital CMV (26% vs 11%, p-value = 0.03), The prevalence of all histologically confirmed malaria was not significantly increased (17% vs 12%, p-value = 0.4). Primiparity (OR 5.3, 95%CI 2.0-13.7), crowding in the compound (defined as living with 13 people or more) (OR 2.9; 95%CI 1.0-8.3), and acute malaria (OR 2.9; 95%CI 1.0-8.4) were found to be independently associated with congenital infection in a multivariable logistic regression (table 2). There were no independent associations between congenital CMV infection and season of birth, maternal age, bednet use, sex of the baby, or maternal body mass index.

**Table 2:** Independent risk factors (OR, 95% CI) for congenital CMV infection

	Crude OR	Adjusted OR
Primiparity	3.2 (1.7–6.1)	5.3 (2.0–13.7)
Crowding in compound	1.7 (0.8–3.4)	2.9 (1.0–8.3)
Acute placental malaria	2.4 (1.0–6.1)	2.9 (1.0–8.4)

Sero-prevalence of CMV infection was assessed at time of delivery among 194 of the mothers of a recruited newborn. All women (100%) had detectable CMV IgG. CMV viral load at the time of delivery was assessed in a subset of 84 mothers, of whom 11 (13.1%) gave birth to a baby with congenital CMV infection. The geometric mean titre (GMT) of the CMV viral load was significantly higher in colostrum (4220 vs 167, p-value = 0.001) in mothers who gave birth to congenitally infected babies, the GMT was borderline significantly higher in vaginal fluids (6000 vs 1107, p-value = 0.05) and not significantly higher in saliva (536 vs 118, p-value = 0.1). There was no association between the GMT of CMV viral load and placental malaria (p-value = 0.6 for the association of active placental malaria with GMT vaginal fluid, p-value = 0.1 with GMT colostrum).

There were no significant differences in clinical evaluation post-partum between the two groups, except for a higher prevalence of hepatomegaly among congenitally infected infants (2.5% vs 0.3%, p-value = 0.03) (table 3). There were no other significant differences apparent at birth, such as in maternal characteristics, sex, birth weight, placental weight, developmental or Apgar score between those with and those without congenital CMV.

**Table 3:** Clinical characteristics at birth of children with and without congenital CMV infection.

	Congenital CMV infection	No congenital CMV infection	p-value
N (%)	40 (5.4)	701 (94.6)	
Jaundice (n, %)	2 (5.0)	14 (2.0)	0.2
Purpura (n, %)	3 (7.5)	46 (6.7)	0.9
Hepatomegaly (n, %)	1 (2.5)	2 (0.3)	0.03
Cardiac murmur (n, %)	0	2 (0.3)	0.7
Neurological score (mean, sd)*	30.6 (2.0)	30.8 (1.8)	0.5
Birth weight (mean, sd)	3058 (489)	3030 (444)	0.7
Birth length (mean, sd)	48.8 (1.9)	48.6 (2.7)	0.6
Head circumference (mean, sd)	34.0 (1.2)	33.8 (2.1)	0.7
Placental weight (mean, sd)	578 (122)	561 (126)	0.4
Apgar (median, IQR)	9 (9–10)	9 (9–10)	0.9

IQR = inter quartile range; sd = standard deviation

Neurological score following Dubrovnivc: maximum score = 35

### Follow up

After the first year of follow up, 13 (1.8%) of the children had died (none of them congenitally infected, p = 0.4), and 66 (8.9%; one of the 40 congenitally infected children, p = 0.1) were lost to follow up, leaving 662 children eligible for clinical and neurological assessment at one year of age. Of these children, the outcome after one year was assessed for 543 children (82.0%) (table 4).

**Table 4:** Clinical characteristics of children with and without congenital CMV infection who already reached one year of age.

	Congenitally CMV infected	Not congenitally CMV infected	p-value
N	40	701	
Routine follow up visits during first year of life (N)	440	8521	
Symptoms reported during monthly follow-up:			
All (%)	53.4	46.6	0.04
Fevers (%)	20.9	18.7	0.2
Cough (%)	16.1	14.7	0.4
Skin problems (%)	9.5	8.9	0.7
Vomiting (%)	5.6	4.4	0.2
Diarrhoea (%)	5.4	5.7	0.8
Died during first year of life (n, %)	0	13 (1.9)	0.4
Lost to follow up (n, %)	1 (2.5)	65 (9.3)	0.1
Available for clinical evaluation at 1 year of age (n, %)	30 (75)	513 (73)	0.9
Weight gain (kg) at 1 year (mean, sd)	5.6 (1.2)	5.7 (1.1)	0.8
Length gain (cm) at 1 year (mean, sd)	24.4 (2.8)	25.1 (2.7)	0.2

During the first year of their life, 8961 follow-up visits to the clinic were made (median of 8 visits per child, range 1–25) during each of which growth and morbidity were routinely monitored. During nearly half of these visits, the mother or caregiver reported some health complaint of the child. Mothers of congenitally infected children reported complaints at 53.4% of the visits compared to 46.6% of visits for those that were not congenitally infected (p-value = 0.04). At one year of age, 30/40 (75.0%) of the congenitally infected and 512/701 (73.0%) of non-congenitally infected children underwent a full paediatric and neurological assessment. No significant differences in developmental, clinical or anthropometric observations were observed between the two groups.

## Discussion

Our data showed that nearly one in every twenty live-born newborns in the study area was congenitally infected with CMV. This is likely to be an underestimate of the actual incidence in the community since children with a low birth weight were initially not included, and some children who were born very ill may have been referred immediately upon delivery and could therefore not be asked to participate in the cohort. Both outcomes may have been associated with congenital CMV infection resulting in an underestimate of the incidence of congenital CMV infection. This prevalence is higher than reported in most other studies, but is consistent with a previous study in The Gambia [21]. This higher prevalence could therefore be related to a higher risk of infection, but it may also be related to under diagnosis in other areas if less sensitive diagnostic methods such as virus culture were used.

No clinical congenital CMV syndrome was observed at birth, and no significant differences in the prevalence of symptoms at birth between those with and without congenital CMV infection. Because recruitment and assessment of CMV status was done at the time of delivery only, no information on (infected) stillbirths is available. This, and the fact that babies who needed an immediate transfer to the referral hospital due to prematurity or serious morbidity were not included either, means that we may have underestimated the clinical impact of congenital CMV infection. A study in Brasil in an area also with a high population CMV seroprevalence observed a similar prevalence of congenital infection in full-term and pre-term babies, with no symptomatic babies among the term-babies, while the majority of the infected pre-term infants were symptomatic [22]. In this cohort, seroprevalence among the subgroup of women assessed at the time of delivery was 100%, which is similar to the seroprevalence found among a group of 93 Gambian children aged 4–5 year old living in the same area (unpublished data). Therefore, it seems highly likely that all or nearly all of the pregnant women will have been infected with CMV prior to the pregnancy, and that most or all of the congenital infections will have resulted from recurrent maternal infection, either through reactivation or reinfection, which is likely to be less severe than infection following a maternal primo-infection [12]. It is also possible that due to high pressure of competing infections [23] non-specific immune factors are stimulated which could contribute to protective immunity. Also, as exposure of the mothers to CMV is high, their natural immunity and control of CMV replication if infected could be better. The significant association of a high maternal viral load with congenital infection indicates that generalised active maternal viral shedding around the time of delivery is related to the risk of the infant becoming infected. Further studies of immune responses to congenital and post-natal CMV infection in this cohort are ongoing.

The finding that the prevalence of placental malaria was higher in congenitally infected infants confirmed our initial hypothesis. We have previously shown that placental infection with *P. falciparum* affects Th1 differentiation of cord blood T lymphocytes [24] and it has been shown before that malaria infection can suppress immunity to a variety of viral infections, such as HIV, Epstein Barr virus, vaccinia and lymphocytic choriomeningitis virus [25], [26], [27]. The production of specific immune factors could inhibit the control of CMV and promote apoptosis of trophoblasts which would facilitate transplacental transfer of CMV, once infection of the cytotrophoblast has occurred [10], and it is possible that placental malaria infection stimulates a similar mechanism leading to an increased transmission. It is also possible that following intrauterine CMV infection, the susceptibility of the placenta to become infected with malaria is increased. Further investigation of this association and possible biological mechanisms involved could give more insights in our understanding of risk factors for congenital infections and could guide interventions to prevent this. An association between placental malaria and primiparity has been found before, which could be associated with a different immune response in primiparous women compared to multiparous women. Perhaps similar mechanisms are involved in the association between placental malaria and congenital infection, as placental malaria was not, and congenital CMV infection was associated with maternal shedding of CMV [28], [29].

We did not assess the HIV status of mothers or babies, since it was considered that stigma still surrounding HIV testing and sero-status would have compromised willingness to participate in the study. Among babies exposed to HIV perinatally, congenital CMV is more common among those who became HIV infected themselves [30]. Co-infection with HIV and CMV was associated with greater immunosuppression and a more rapid clinical progression compared to children infected with HIV only [31]. HIV-1 prevalence among pregnant women in The Gambia is estimated to be around 1.0% and HIV-2 at 0.8% [32]. A previous study from Brasil in a highly endemic CMV area reported similar congenital CMV prevalences in HIV infected and uninfected mothers [33]. Thus, it seems unlikely the lack of these data will have biased our results on transmission risks and outcome. We could not confirm previous reports which found an increased risk for females versus males for adverse outcome following congenital CMV infection [34].

The prevalence of reported morbidity during follow-up tended to be higher in the congenitally infected group, but this did not reach statistical significance. It is clear from this study that in spite of ongoing urbanisation, and increasing availability of health facilities, the burden of illness among infants and young children in developing countries remains very high. At nearly half of all routine visits at home, one or more complaints were reported. The relatively low mortality amongst the infants is in stark contrast to the high percentage of mothers who reported the death of a previous child, and might be associated with a study bias due to the close follow up and continuous medical care offered by the study team. Although mothers of congenitally infected children tended to report slightly more complaints, this was only of borderline significance. A previous study among immunocompetent patients infected with CMV also found a high proportion of general malaise complaints rather than any specific clinical correlates [35]. Hearing loss, often progressive, may be the only specific complication among those with an asymptomatic congenital infection [36]. It is possible that if we had been in a position to use advanced electronic stimulations methods for detecting early hearing loss, such as auditory brainstem response or otoacoustic emissions, we would have been able to assess if any early hearing loss differences existed (37). The mean age at which hearing loss is diagnosed among congenitally infected children following a non-primary infection of the mother was estimated at 39 months [38]. Continued follow up of this cohort is needed to find out whether or not a specific clinical impact becomes apparent around four to five years of age when a comprehensive neurological and auditory examination is planned. Nevertheless, it is also possible that we did not observe major differences in outcome during the first year of life for children born with a congenital CMV infection. This could be due to the fact that other environmental factors, and/or other early life infections had a much larger impact on health in this resource-poor, semi-urban, community, which obscured more modest effects of the congenital infection; or through interaction did not reflect in the overall outcome indicators so far.

In recent years, antiviral therapy has become available to mitigate the risk of clinical complications following congenital CMV infections [39], but apart from the serious potential side effects, this option may not be affordable to populations in low income countries. In the long run, a (maternal) vaccine which protects against primary and recurrent disease will be a better and more affordable option to reduce the morbidity associated with congenital CMV infection [40]. In the mean time, improved understanding of risk factors associated with congenital infection and with adverse outcome following infection is needed in order to develop and test effective interventions.
